# HIV Risks, Testing, and Treatment in the Former Soviet Union: Challenges and Future Directions in Research and Methodology

**DOI:** 10.5195/cajgh.2015.225

**Published:** 2016-01-04

**Authors:** Victoria M. Saadat

**Affiliations:** Department of Health Research and Policy, Stanford University of Medicine, Stanford, CA

**Keywords:** HIV, HIV treatment, HIV testing, barriers, sex workers, literature review, Central Asia, Russia

## Abstract

**Background:**

The dissolution of the USSR resulted in independence for constituent republics but left them battling an unstable economic environment and healthcare. Increases in injection drug use, prostitution, and migration were all widespread responses to this transition and have contributed to the emergence of an HIV epidemic in the countries of former Soviet Union. Researchers have begun to identify the risks of HIV infection as well as the barriers to HIV testing and treatment in the former Soviet Union. Significant methodological challenges have arisen and need to be addressed. The objective of this review is to determine common threads in HIV research in the former Soviet Union and provide useful recommendations for future research studies.

**Methods:**

In this systematic review of the literature, Pubmed was searched for English-language studies using the key search terms “HIV”, “AIDS”, “human immunodeficiency virus”, “acquired immune deficiency syndrome”, “Central Asia”, “Kazakhstan”, “Kyrgyzstan”, “Uzbekistan”, “Tajikistan”, “Turkmenistan”, “Russia”, “Ukraine”, “Armenia”, “Azerbaijan”, and “Georgia”. Studies were evaluated against eligibility criteria for inclusion.

**Results:**

Thirty-nine studies were identified across the two main topic areas of HIV risk and barriers to testing and treatment, themes subsequently referred to as “risk” and “barriers”. Study design was predominantly cross-sectional. The most frequently used sampling methods were peer-to-peer and non-probabilistic sampling. The most frequently reported risks were condom misuse, risky intercourse, and unsafe practices among injection drug users. Common barriers to testing included that testing was inconvenient, and that results would not remain confidential. Frequent barriers to treatment were based on a distrust in the treatment system.

**Conclusion:**

The findings of this review reveal methodological limitations that span the existing studies. Small sample size, cross-sectional design, and non-probabilistic sampling methods were frequently reported limitations. Future work is needed to examine barriers to testing and treatment as well as longitudinal studies on HIV risk over time in most-at-risk populations.

## Historical overview

The Former Soviet Union (FSU)—a group of countries and republics spread out across a vast area spanning Europe and Asia—is harboring one of the fastest growing epidemics of Human Immunodeficiency Virus/Acquired Immune Deficiency Syndrome (HIV/AIDS) in the world.^1^ Little attention, however, was paid to the epidemic during its early years. The epidemic’s growth was masked by low infection rates throughout the region and the pressure felt by each newly formed nation to establish new and independent political and economic infrastructures before addressing public health issues. What remains today is a low-profile, yet alarming, spread of HIV that necessitates a strategic response. Large parts of the FSU, especially Central Asia, have been experiencing one of the fastest-growing epidemics of HIV in the world.^2^ Some areas have recorded infection rates doubling every year since 2000, when steady record keeping began.^3^ Another study demonstrated a 13% increase in new HIV infections in Central Asia and Eastern Europe between 2006 to 2012.^4^ Research into the causes of the epidemic and the barriers to its alleviation is the crux of an effective strategy.

When the Soviet Union collapsed in 1991, the constituent republics (Russian Federation, Ukraine, Uzbekistan, Kazakhstan, Belarus, Azerbaijan, Georgia, Armenia, Tajikistan, Moldova, Kyrgyzstan, Lithuania, Turkmenistan, Latvia, and Estonia) were left to transition to independence with limited resources and guidance. During the transition period, economic collapse and political turmoil catalyzed many societal changes.^5^ Economic transition severely weakened public health infrastructure due to loss of funding from state subsidies, widespread unemployment, and an increase in private practice—where fee-for-service and hidden payments became the norm.^6^ After the healthcare infrastructure became stagnated and devoid of necessary funding, physicians and researchers began to notice a rise in HIV prevalence. The spread of HIV also coincided with a decline in life expectancy, higher levels of alcohol and injection drug use (IDU), and increased rates of co-infection with tuberculosis (TB), hepatitis C virus (HCV), and syphilis among other sexually transmitted infections (STI).^7^,^8^

## Driving forces behind the epidemic

Efficacy of HIV testing and access to treatment have been evaluated at both the individual and societal levels in many regions of the world, but studies of how vulnerable populations—especially IDU—access these services in the FSU have been limited.^9^ In recent years, approaches to HIV/AIDS worldwide have broadened to focus not only on individual risk-taking behavior, but also on the environmental and societal factors that influence risky behavior and use of health services.^10^,^11^ Most-at-risk populations—IDU, migrant workers, and commercial sex workers (CSW)—are particularly vulnerable without access to HIV testing, treatment, and prevention resources. They also are among the FSU’s least studied groups.^10^ Initiating public health research around highly stigmatized populations, however, has proven to be especially challenging in the FSU.^12^ In the most extreme case, this stigma has resulted in little to no research on HIV in Turkmenistan, where it is unlawful to diagnose or report a patient with HIV.^13^,^14^ This review makes little reference to the HIV situation in Turkmenistan, where there is limited national data.

Furthermore, the body of literature on HIV in the FSU is just beginning to take shape. However, the eventual goal of building a substantive body of literature around the causes of and barriers to reduction of HIV is to identify why at-risk populations are more vulnerable to HIV, as well as the barriers underlying suboptimal access to testing and treatment.^15^ Knowing these barriers, programs can be redirected and new initiatives prepared. Furthermore, in order to conduct more effective research, challenges and limitations of past studies must be discussed. The primary aim of this review is to systematically evaluate the literature and provide a concise review of research and methodological challenges to-date on the HIV epidemic in the FSU. A second goal of this analysis is to provide guiding factors for the planning and implementation of future studies for the design of more effective testing and treatment programs in the region.

## Methods

### Search methods

Search terms used on PubMed included “HIV”, “AIDS”, “human immunodeficiency virus”, “acquired immune deficiency syndrome”, “Central Asia”, “Kazakhstan”, “Kyrgyzstan” “Uzbekistan”, “Tajikistan”, “Turkmenistan”, “Russia”, “Ukraine”, “Armenia”, “Azerbaijan”, and “Georgia” (Countries were chosen on the basis of availability of research literature). Bibliographies of relevant articles and reviews were scanned for further studies.

**Figure 1.**
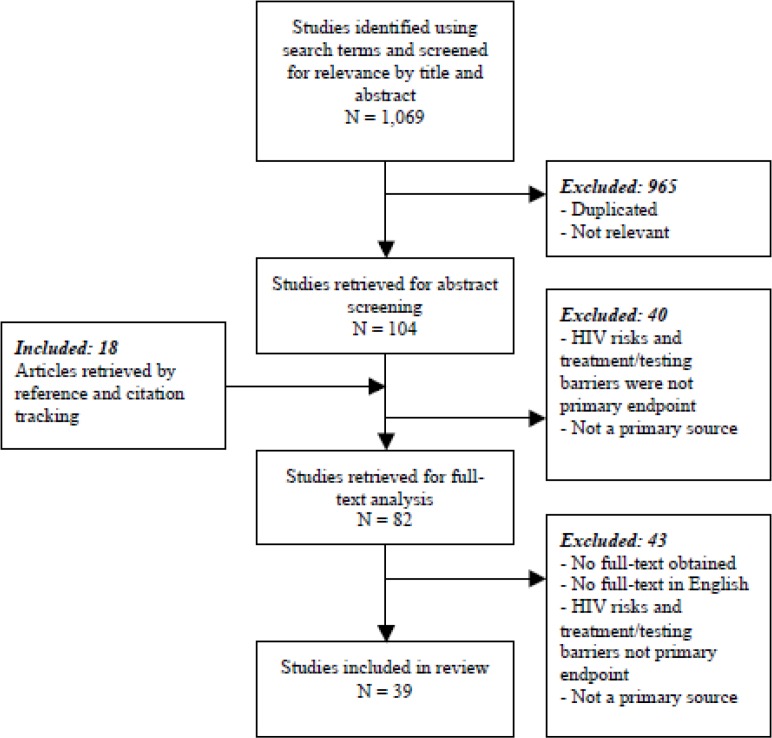
**Flow chart depicting the review process of selecting studies for inclusion and analysis of manuscripts examining risks of HIV infection, barriers to HIV treatment and challenges in HIV prevention**

To be eligible, studies had to be published in English, contain primary data, and identify HIV risk factors and/or barriers to HIV testing or treatment as primary outcomes. These overarching themes are subsequently referred to as “risk” and “barriers”. Studies highlighting risks and barriers were chosen for review because HIV prevention and treatment efforts cannot be realized unless the underlying risks are understood.^16^ The flow of study selection is illustrated in Figure 1. The search engine PubMed was used in the collection of studies for this review. Studies deemed irrelevant were either only tangentially related to HIV in the former Soviet Union or contained the key words but were not answering a research question that contributed to the aims of the review.

### Data extraction

Table 1 shows the main features of each study; Tables 2a, 2b, and 2c summarize the most frequently reported risks, barriers to testing, and barriers to treatment, respectively.

## Results

### Study location

The literature collected was sorted into geographical categories by country. The number of studies from each are as follows: Armenia (n=3)^17^–^19^; Azerbaijan (n=2)^20^,^21^; Georgia (n=2)^22^,^23^; Kazakhstan (n=6)^24^–^29^; Kyrgyzstan (n=3)^30^–^32^; Russia (n=13)^33^–^45^; Tajikistan (n=5)^46^–^50^; Ukraine (n=3)^32^,^51^,^52^; and Uzbekistan (n=2)^53^,^54^. Studies were most heavily represented in Russia (33% of studies), Kazakhstan (15%), and Tajikistan (13%).

### Study design

The majority of studies employed a cross-sectional design (n=36). Of these studies, 10 also obtained biological samples to determine HIV status of participants. The only longitudinal study was from Georgia where investigators and implemented both HIV testing and biobehavioral surveys at two different time points, three years apart from each other.^23^ However, the study reported that the sample size was insufficient to power a comparison between the two time points. Therefore, small sample size among a marginalized population was a key limiting factor.

### Study population

IDU were the target population in 17 studies (44%) and were conducted in each country except Turkmenistan. Other major populations were migrant workers (n=8), female and CSW (n=7).

### Sampling methods

The most common sampling methods were purposive sampling (n=19), convenience sampling (n=6), and respondent-driven sampling (includes snowball sampling) (n=5).

### Study limitations

The most frequently reported methodological challenges were cross-sectional study design (n=12), inability to obtain a representative sample (n=11), use of self-report (n=11), sub-optimal participant recruiting procedures (n=8), and/or a small sample size (n=6). Further methodological limitations included: data were found not to be generalizable outside of the country in which the research was conducted (n=5), specifically having used purposive or snowball sampling to recruit participants (n=5), low participation rates (including not having obtained data on specific groups that declined to participate) (n=4), translation issues and cultural misunderstanding of qualitative data (n=4), and likely underreporting of risky, illegal, and/or stigmatized behaviors in surveys and interviews (n=4).

### Risk factors for HIV infection

The most frequently reported categories of risks were condom misuse (n=9), risky intercourse (n=9), unsafe injection practices among IDU (n=8), and spread of infection through people who inject drugs (n=8) (Table 2). Additional groups of risk factors included migration challenges (n=6), low HIV/AIDS knowledge (n=4), and a history of STI (n=4).

### Barriers to HIV testing

The most prominent barriers to testing for HIV status included the perception that it was shameful to test for HIV (n=2), that testing was inconvenient (n=2), and that test results would not be held confidential (n=2) (Table 3).

### Barriers to HIV treatment

The most frequently reported barriers to obtaining treatment for HIV were based on a distrust in the treatment system and experience with the lack of efficiency in the structure of the treatment system (Table 4). Specifically, the barriers included a fear of disclosure of treatment status (n=6), an inefficient and ineffective treatment structure (n=6), difficulty in registering for and/or being accepted into a treatment facility (n=5), and difficulty in accessing treatment facilities (n=5).

## Discussion

This is, to our knowledge, the first systematic review of the current body of research spanning the stages of HIV infection in the FSU, from risk/infection through testing and treatment. The results reveal several important areas in which the current state of research and knowledge is incomplete due to methodological limitations of many studies. As shown in Tables 3 and 4, barriers to testing and treatment remain strong among at-risk populations (IDU, CSW, and migrant workers) but remain inadequately researched when compared to the number of studies examining risk factors for HIV infection. Among the reasons for this include sample recruitment challenges, and other methodological challenges, which are further discussed below.

### Most-at-risk populations

CSW, men who have sex with men (MSM), IDU, and migrant workers have been found to be key players in the spread of HIV.^1^,^4^,^7^ However, they are the most stigmatized and marginalized groups and have very little access to HIV treatment.^55^ Further, economic, social, and institutional factors in the region can be linked to the spread of HIV among these groups.

### Migrant workers

Migration between Russia, the Caucuses, and Central Asia has been observed as a driver of the epidemic.^1^,^11^,^56^ Many Tajik and Kyrgyz migrants travel through Kazakhstan and into Russia to find work. As might be expected, extensive travel often puts them at risk.^56^ Often, financially compromised and separated from family, migrants have been shown to engage in behaviors that increase the risk of HIV transmission.^57^ Their financial and legal status in the host country make it extremely difficult to access medical care should HIV be suspected or treatment needed.^9^

### Injection drug users

Throughout the FSU, the IDU population has been growing and is associated with harmful drug use and co-infections of TB and HCV.^4^ The example of Central Asia demonstrates both behavioral, economic, and geographical factors, among others, at play in the concentration of HIV among IDU.^58^–^60^ The trafficking of opium out of Afghanistan results in large amounts of the drug being transported through Kazakhstan, fueling rapid growth of the nation’s population of IDU. Additionally, Kazakhstan and other Central Asian nations’ location at the centers of labor migration routes compound the effect of drug trafficking: when migratory patterns considerably overlapped with drug trafficking routes, the number of cases among IDU increased five-fold in the 13 years following Kazakhstan’s independence.^61^

### Commercial sex workers

This group consists of both men and women who engage in sex work for compensation and suffer tremendously from the stigma that accompanies their work. Among this group, female sex workers are more stigmatized than their male counterparts. Those who also inject drugs experience a form of double jeopardy.^62^

### Methodological challenges

One, studies have been conducted in many, but not all, of the countries of the FSU. Research in the field of HIV/AIDS is particularly limited in Ukraine and Georgia, along with being severely limited in Turkmenistan. While the number of studies from Russia and Kazakhstan, for instance, are relatively numerous, they cannot necessarily be generalized to other FSU countries. This could be explained by the simultaneous similar-and-different nature of the countries: on one hand, they shared some common elements of their political, social, and economic history for most of the 20^th^ century. On the other hand, each country has its own history and ethno-cultural fabric, which is likely to uniquely affect the mentality and psychology of its people. Therefore, studying the risks and barriers within every FSU country is necessary in order to help each one best prepare and implement an approach to ameliorate the HIV/AIDS epidemic.

Two, many of the studies used a cross-sectional design in collecting their data. It is difficult to make statements of causation from such designs. More longitudinal designs are needed to study the range of factors for any given at-risk group. For example, migrant workers may need to be studied throughout the migration process to evaluate the stage of the migration experience that introduces the most vulnerability to exhibit HIV risk behaviors.

Three, many of the studies employed non-probabilistic sampling. It is difficult to know the probability with which the target population has been represented in the sample when using a non-probabilistic method of sampling. Such methods that have been employed in this review’s studies include convenience sampling, purposive sampling, and snowball sampling. The studies required participation by individuals who exhibit illicit, illegal, or stigmatized behaviors and are, therefore, socially marginalized. Convenience sampling, purposive sampling, and snowball sampling were used to gain access to such populations. Although migrant workers, IDU and CSW are understandably difficult to access and representatively sample, studies could be designed in a more rigorous way that takes into account these limitations of working with hard-to-sample populations that are hidden and lack most formal forms of rosters or lists of documentation, from which probabilistic samples could be obtained. In a separate search of the literature about HIV risk behavior studies in other parts of the world, including Thailand and Australia, it was interesting to find that many of the studies did not demonstrate a need for purposive or respondent-driven sampling techniques, for instance. Instead, the researchers often approached CSW, in many cases, in testing or treatment facilities.^63^,^64^ This may likely indicate a difference in the difficulties inherent in recruiting marginalized populations in the FSU, when compared to the same task in other parts of the world.^65^

Four, many of the studies reported that it was likely that subjects may have underreported stigmatized, illegal, or risky behaviors in self-report questionnaires and interviews. Although not verified, it was an observation made by researchers who were likely aware of the stigmatized nature of most-at-risk populations. For this reason, it was thought to be likely, given that study participants may have distrusted the researchers and feared that the interview results deemed confidential would be released to the police.^66^ Furthermore, data obtained from self-report can be subject to the “social-desirability bias,” by which a participant may answer questions in a certain manner in order to portray themselves as lawful and socially acceptable. While this insight is helpful in interpreting the data, it sill reveals that the data acquired are not thoroughly accounting for the range and prevalence of behaviors that put subjects at risk for HIV infection or pose barriers to testing and treatment.

### Limitations

There are several limitations of the review that must be noted. First, while most of the studies were generally accessible in English, several studies were available only in Russian (n=8) or full-text was inaccessible. Without the ability to identify and retrieve all relevant studies, the review’s scope may diminish from the ideal. Second, the study of HIV is a relatively new area of epidemiologic and public health focus in the FSU, and thus the number of relevant studies is limited, resulting in 39 eligible studies for review. Third, the methodological limitations that were extracted from the study manuscripts and tabulated above were based on what the authors had listed in their own evaluation of study limitations and/or from what was available in the methodological descriptions of the studies. For instance, purposive sampling—as a methodological limitation in a study—was determined from the methodological descriptions and/or from the discussion of limitations provided by the study’s authors. This means that certain methodological limitations deemed infrequent in this review—especially “distrust of researchers” (3% of studies reviewed)—should not be viewed as certainly infrequent. Many of the studies may have suffered from participant distrust, which may have either gone unnoticed and had an effect on data or sampling outcomes or have been noticed but not reported in the manuscript. Participant distrust of the researchers can have an effect on many parts of a study that involve most-at-risk populations: participation rate, sample size, and underreporting can all be affected, but it is important to note in FSU-based studies when distrust occurs in order to help determine ways to improve the relationship between most-at-risk populations and researchers.

## Conclusion

### HIV research in the former Soviet Union

Today, the epidemic grows as risky behavior continues, and prevention and treatment programs face difficulties gaining a foothold in the still-transitioning atmosphere of the FSU. However, the findings of this review reveal a few particular ways in which the current state of knowledge is incomplete as a result of methodological limitations of many of the existing studies. For instance, while 20 studies reported risk factors for HIV infection, only seven of the 39 studies reported barriers to HIV testing, and 9 studies reported barriers to treatment. These numbers illustrate where the bulk of the research has been conducted in the HIV infection pathway (risks factors and infection, testing, and treatment) in the FSU. One reason for this finding may be that there are many more individuals at risk for or infected with HIV than there are individuals who have sought testing and/or treatment. Given that small sample size has been a limitation and concern among many of the reviewed studies, it may be a reason for the relative scarcity of studies on barriers to testing and treatment when compared to studies on the risk factors for HIV infection.

### Recommendations for future studies

Going forward, energy and resources would be best spent on research to study the barriers to getting tested and treated for HIV. Of the studies included, the number of which that looked barriers to testing and treatment was minimal compared to what was aimed at studying the risks of HIV infection. Future research would include a combination of studies that address the described methodological challenges and one or more attempts at meta-analysis of the data from thematically aligned studies. Efforts to apply the results from the above-mentioned research would assist in improving existing HIV programs and advising the development of new ones in the FSU.^67^,^68^

## Figures and Tables

**Table 1:** Summary of studies examining risks of HIV infection, barriers to HIV treatment and challenges in HIV prevention. Pertinent characteristics include location, study aim, design, population, sampling method(s), and sample size

**Reference**	**Location**	**Aims**	**Outcome Methods**	**Population**	**Sampling Method**	**Sample**
Markosyan et al. (2007)	Armenia	Describe HIV risk/preventive behaviors and correlates among Armenian FSW	Questionnaire, focus groups, interviews	Armenian FSW	Convenience sample	98
Lang et al. (2013)	Armenia	Identify the association of gender-based violence with sexual risk among FSW	Survey	Armenian FSW	Convenience and snowball sampling	120
Johnston et al., (2014)	Armenia	Present risk behavior associations for HIV and HCV infection among PWID	Interview and testing for HIV and HCV	PWID injecting drugs within the past three months	RDS	270
Nassibov et al., (2005)	Azerbaijan	Examine the prevalence and context of injection drug use and HIV-risk behaviors and trends in HIV transmission	Survey and focus groups	IDU and key informants (medical staff, police, and legal experts)	Not described	400
Botros et al., (2009)	Azerbaijan	Assess HIV prevalence and associated risk behaviors among truck drivers	Questionnaire and sero-surveillance blood testing	Truck drivers traveling through Azerbaijan	Convenience sampling	3,763
Otiashvili et al. (2013)	Georgia	Investigate the factors that may facilitate or hinder substance-using women’s help-seeking behavior or access to treatment services	Secondary analysis of in-depth interviews	Substance-using women and providers of health-related services	Word-of-mouth	89
Tsereteli et al. (2013)	Georgia	Investigate HIV testing practice among FSW and MSM and to identify determinants of never testing behavior among MSM	Bio-behavioral surveys	FSW and MSM in Tbilisi, Georgia	FSWs were recruited through time-location sampling; Recruitment of MSM was carried out through RDS	278
Viale, BN (2010)	Kazakhstan	Assess perceived barriers to seeking and accessing voluntary testing	Survey	IDU in Kazakhstan	RDS	1,071
El-Bassel et al. (2014)	Kazakhstan	Compare FWID and females who do not inject drugs, examining associations between history of IDU and HIV and HCV risk behaviors	Reanalysis of data from prior RCT with self-reported responses and biological assays	Female partners of MWID: Both FWID and non-injecting female partners	Trained research assistants recruited potential study participants from neighborhoods where IDU gather as well as HIV clinics and needle exchange programs (for more detail see El-Bassel et al., 2013)	364
El-Bassel et al. (2011)	Kazakhstan	Examine associations between mobility patterns and HIV risks among male and female migrant market vendors	Structured interview	Male and female migrant market vendors in Almaty	Randomized sampling using GIS (mapping of stalls at Barakholka)	422
El-Bassel et al. (2013)	Kazakhstan	Examine associations between HIV serostatus, socio-demographic factors, and sexual and drug risk behaviors	Self-report data and biological assays for HIV serostatus	IDU and their heterosexual intimate partners	Word-of-mouth and targeted outreach in known neighborhood locations where IDU gather	728
Berry et al. (2012)	Kazakhstan	Measure HIV risk factors and HIV prevalence among MSM	Questionnaire and HIV test	MSM in Almaty	RDS	400
Boltaev et al. (2012)	Kazakhstan	Evaluate the quality and effectiveness of the MAT pilot in Kazakhstan and review implementation	In-depth qualitative interview	MAT patients in Kazakhstan	Not specified	93
Deryabina, A. (2011)	Kyrgyzstan	Understand the current status of HIV services for most-at-risk populations (MARP), access to, and quality of services provided	Semi-structured interviews and focus groups	IDU, CSW, former prisoners, and leaders of various HIV/AIDS groups in Chui Oblast and Bishkek City	MARP representatives who participated in FGDs or individual interviews were recruited through NGO representatives (outreach workers) and thus only included clients of HIV-related services	243
Messner et al. (2013)	Kyrgyzstan	Identify gender-based constraints to accessing HIV/AIDS programs and services	Interviews	Individuals from various governmental organizations	A list of key informants was developed in collaboration with USAID/Kyrgyzstan and additional interviewees were identified during the in-country data collection	60
Spicer et al. (2011)	Kyrgyzstan	Explore access barriers to HIV/AIDS services experienced by a key risk group of IDU	Semi-structured interviews	IDU and stakeholders in Ukraine and Kyrgyzstan	Purposive sampling	228
Tkatchenko-Schmidt, et al. (2008)	Russia	Examine attitudes of Russian policy-makers and HIV stakeholders towards HR scale up	Semi-structured interviews	HIV-focused governmental organizations and NGOs in Volgograd	Purposive and chain sampling	58
Bobrova et al. (2006)	Russia	Assess factors that impact IDU access to treatment	Qualitative interviews	IDU	Purposive sampling	86
Sarang et al. (2008)	Russia	Investigate IDU access to needles and syringes	Qualitative interviews	IDU in Moscow, Volgograd, Barnaul	Targeted and snowball sampling	209
King et al. (2013)	Russia	Determine facilitators of and barriers to accessing HIV services	In-depth qualitative interviews	FSW in St. Petersburg	Purposive sampling	29
King et al. (2013)	Russia	Better understand how stigma and discrimination influence HIV service utilization	Questionnaire	FSW	Purposive sampling	139
Vasquez et al. (2013)	Russia	Define characteristics and barriers to HIV care	Questionnaire	People receiving HIV treatment in St. Petersburg	Convenience sampling	152
Sarang et al. (2013)	Russia	Explore barriers to accessing ART among PWID	In-depth qualitative interviews	PWID in Yekaterinburg	Purposive sampling	42
Zabrocki et al. (2013)	Russia	Understand socio-structural barriers, protective factors, and HIV sexual risk	Interviews	Unmarried female migrants in Moscow	Purposive sampling	30
Niccolai et al. (2010)	Russia	Estimate HIV prevalence and testing patterns among IDUs	HIV and STI testing; Survey	IDU in St. Petersburg	RDS	387
Kruse et al. (2009)	Russia	Examine behaviors associated with HIV risk among IDUs	Questionnaire and HIV testing	IDU	Purposive sampling	900
Amirkhanian et al. (2011)	Russia	Explore health service access of persons living with HIV	Questionnaire	Individuals with HIV/AIDS from 5 St. Petersburg health care and social service agencies	Convenience sampling	470
Amirkhanian et al. (2011)	Russia	Determine how well migrant workers understand HIV risk factors and behaviors that increase HIV risk	Questionnaire and survey	Male labor migrants in St. Petersburg	Convenience sampling	499
Weine et al. (2008)	Russia	Characterize HIV/AIDS risk; Identify contextual factors that could impede or facilitate a preventive intervention	Ethnographic interview and survey	Tajik male migrant workers in Moscow	Purposive sampling at work sites	30
Stachowiak et al. (2006)	Tajikistan	Examine differences by ethnicity of HIV prevalence and correlates among IDU	Questionnaire and HIV testing	Active adult IDUs	Purposive sampling	489
Beyrer et al. (2009)	Tajikistan	Determine HIV, HCV, and syphilis prevalence and correlates	Survey; HIV, HCV, and syphilis testing	Active adult IDUs	Purposive sampling	491
Weine et al. (2012)	Tajikistan	Determine the role of trauma and PTSD symptoms in the context of migration-associated HIV risk behaviors	Survey	Tajik married male labor migrants in Moscow	Probabilities proportionate to size (PPS) methods; simple random sampling (SRS)	400
Golobof et al. (2011)	Tajikistan	Understand labor migrants’ wives’ knowledge, attitudes, and behaviors regarding HIV/AIDS risk and protection	Minimally structured interviews and field observations	Tajik wives in Dushanbe married to male migrant workers in Moscow	Purposive sampling	30
Jing et al. (2012)	Tajikistan	Investigate powerlessness in HIV risk among internal and external male labor migrant workers from Tajikistan	Minimally structured interviews and field observations	Migrants working in Regar; Migrants working in Moscow	Purposive sampling	60
Mimiaga et al. (2010)	Ukraine	Examine barriers and facilitators to HAART adherence	Semi-structured focus groups	HIV-infected IDU seeking treatment at the City AIDS Center, Kiev	Purposive/convenience sampling: Participants recruited from those attending treatment the AIDS Center	16
Booth et al. (2013)	Ukraine	Learn how experiences with the legal system (police and courts) correlate with HIV among IDU	Semi-structured interviews	IDU; police and members of the court	Not specified	19
Spicer et al. (2011)	Ukraine	Explore multiple access barriers to HIV/AIDS services experienced by a key risk group of IDU	Semi-structured interviews	IDU (current and former) and national and sub-national stakeholders in Ukraine and Kyrgyzstan	Purposive sampling: Client interviewees were recruited with the agreement of HIV/AIDS service providers who introduced potential interviewees to the researchers	391
Todd et al. (2007)	Uzbekistan	Examine condom use and HIV testing use among FSW	Questionnaire, interview, and HIV testing	FSW in Tashkent	Purposive sampling by outreach workers affiliated with Istiqbolli Avlod, a NGO in Tashkent	448
Sanchez et al. (2006)	Uzbekistan	Determine HIV prevalence and potential associations with sociodemographic and behavioral factors among IDU	Survey and HIV biosurveillance	IDU in Tashkent	Purposive sampling by the Center for AIDS Prevention and Control and in IDU gathering locations	701

**Table 2.** Risk factors for infection with HIV as determined by the reviewed studies with detailed aspects as well as the supporting studies

**Risk Factor**	**Reported Details**	**Supporting Studies**
Condom use	Irregular, inconsistent, and incorrect use of condoms	Beyrer et al. (2009), Botros et al. (2009), Lang et al. (2013), Amirkhanian et al. (2011), Sanchez et al. (2006), Markosyan et al. (2007), Vasquez et al. (2013), Zabrocki et al. (2013), Jing et al. (2012)
Risky intercourse	Sex with IDU clients, MSM status, unprotected sex, unprotected sex with CSW, earlier age of initiation of sex work, transactional sex, multiple female partners in last 3 months, having unprotected anal intercourse with male partners, unprotected receptive anal sex	Markosyan et al. (2007), Amirkhanian et al. (2011), Lang et al. (2013), Weine et al. (2008), Lang et al. (2013), Berry et al. (2012), Amirkhanian et al. (2011), Berry et al. (2012), Berry et al. (2012)
Unsafe injection practices	Daily injecting, injecting alone, starting injecting at a younger age of initiation of illegal drug use, longer history of drug abuse, rushed injections due to fear of the police, IDU status, being female IDU	Stachowiak et al. (2006), Beyrer et al. (2009), Vasquez et al. (2013), Booth et al. (2013), Vasquez et al. (2013), Amirkhanian et al. (2011), El-Bassel et al. (2014), El-Bassel et al. (2011)
Migration challenges	Being Tajik or Uzbek nationality, frequent travel outside of current place of residence, harsh living and working conditions, lack of legal protection from the government, poor social support	Beyrer et al. (2009), El-Bassel et al. (2013), El-Bassel et al. (2013), Weine et al. (2008), El-Bassel et al. (2013), Amirkhanian et al. (2003)
Threats from police	Police planting drugs, IDU paying police to avoid arrest, prior confiscation of pre-filled syringes, history of incarceration	Booth et al. (2013), Booth et al. (2013), Booth et al. (2013), Booth et al. (2013), El-Bassel et al. (2014)
Low HIV/AIDS knowledge	Incomplete or vague knowledge of HIV transmission	Jing et al. (2012), Markosyan et al. (2007), Zabrocki et al. (2013), Amirkhanian et al. (2011)
History of STI	Current STI symptoms, history of STI, prior history of hepatitis	Berry et al. (2012), Botros et al. (2009), Sanchez et al. (2006), Lang et al. (2013)
Concurrent alcohol and drug use	Drinking alcohol, non-injection drug use	Jing et al. (2012), Markosyan et al. (2007), Berry et al. (2012)
Pressure not to use condom	Fear of sexual partners’ reaction to condom use	Lang et al. (2013), Golobof et al. (2011)
History of drug abuse treatment	History of undergoing drug abuse treatment multiple times	Stachowiak et al. (2006), Beyrer et al. (2009)

**Table 3.** Barriers to HIV testing as determined by the reviewed studies detailed aspects of each category and supporting studies

**Barrier to Testing**	**Reported Details**	**Supporting Studies**
Shame	Thoughts that testing is shameful, HIV stigma	Golobof et al. (2011), King et al. (2013)
Convenience of testing	Low access and hard to find testing locations, inconvenient clinic hours	Tsereteli et al. (2013), Viale, BN (2010)
Confidentiality of testing	Fear of being disclosed as an IDU, fear that testing results would not remain confidential	Nassibov et al. (2005), Tkatchenko-Schmidt, et al. (2008)
Fear of result	Fear of a positive test result	Viale, BN (2010)
Priorities	Perception that more immediate problems take priority	Viale, BN (2010)
Self-perception of HIV risk	Considering self at low or no risk for HIV	Tsereteli et al. (2013)
Lack of experience in sex work	Engaging in sex work less than 2 years, younger than 21, initiated sex work at the age of 18 or younger	Todd et al. (2007)

**Table 4.** Barriers to HIV treatment as determined by the reviewed studies with detailed aspects of each and a list of the supporting studies for each category

**Barriers to treatment**	**Reported details**	**Supporting studies**
Fear of disclosure	Lack of anonymity/confidentiality of treatment, fear of registration as IDU, fear of police around treatment centers, criminalization of drug use at treatment centers, harassment and discrimination by police	King et al. (2013), Bobrova et al. (2006), Otiashvili et al. (2013), Sarang et al. (2008), Spicer et al. (2011), Mimiaga et al. (2010)
Inefficient and ineffective treatment structure	Shortages of commodities and human resources, low knowledge and skills of service providers, insufficient drug policies, limited opportunities for staff development, complexity of drug treatment regimen, services and entitlements	Spicer et al. (2011), Otiashvili et al. (2013), Otiashvili et al. (2013), Boltaev et al. (2012), Mimiaga et al. (2010), Spicer et al. (2011)
Difficult to register for or be accepted into treatment	Bureaucracy, tough registration system, organizational barriers, lack of legal status while being a migrant worker	Sarang et al. (2008), King et al. (2013), Spicer et al. (2011), Zabrocki et al. (2013), King et al. (2013)
Difficult to access treatment facilities	Scarce infrastructure of narcological facilities, inadequate access and coverage, insufficient supply management, geographic proximity and access, lack of availability of comprehensive treatment programs, restrictive methadone dispensing policies	Boltaev et al. (2012), Boltaev et al. (2012), Boltaev et al. (2012), Otiashvili et al. (2013), Boltaev et al. (2012)
Unable to afford treatment	Financial constraints (especially for migrant workers)	Zabrocki et al. (2013), Bobrova et al. (2006), Otiashvili et al. (2013), King et al. (2013)
Stigma	Stigmatization of HIV/AIDS and drug use, discrimination among government service providers	Spicer et al. (2011), Mimiaga et al. (2010), Spicer et al. (2011), Bobrova et al. (2006)
Distrust in treatment	Lack of belief in treatment effectiveness, perceived low efficacy, feeling that harm reduction programs are forced on them from outside	Tkatchenko-Schmidt, et al. (2008), Bobrova et al. (2006), Tkatchenko-Schmidt, et al. (2008)
Drug use policies in treatment	Opioid dependence, fear of treatment being withheld b/c drug use	Mimiaga et al. (2010), Sarang et al. (2008)
Limited knowledge	Limited knowledge of HIV/AIDS risk factors	Spicer et al. (2011)
Mental Health Problems	Co-morbid mental health problems	Mimiaga et al. (2010)

## References

[1] Donoghoe MC, Lazarus JV, Matic S (2005). HIV/AIDS in the transitional countries of Eastern Europe and Central Asia. Clin Med.

[2] Thorne C, Ferencic N, Malyuta R, Mimica J, Niemiec T (2010). Central Asia: Hotspot in the worldwide HIV epidemic. Lancet Infect Dis.

[3] Parfitt T (2003). Drug addiction and HIV infection on rise in Tajikistan. Lancet.

[4] DeHovitz J, Uuskula A, El-Bassel N (2014). The HIV Epidemic in Eastern Europe and Central Asia. Curr HIV/AIDS Rep.

[5] Atlani L, Caraël M, Brunet JB, Frasca T, Chaika N (2000). Social change and HIV in the former USSR: The making of a new epidemic. Soc Sci Med.

[6] Balabanova D, McKee M, Pomerleau J, Rose R, Haerpfer C (2004). Health service utilization in the former soviet union: Evidence from eight countries. Health Serv Res.

[7] Walsh N, Maher L (2013). HIV and HCV among people who inject drugs in Central Asia. Drug Alcohol Depend.

[8] Atun R, Olynik I (2008). Resistance to implementing policy change: The case of Ukraine. Bull World Health Organ.

[9] Terlikbayeva A, Zhussupov B, Primbetova S (2013). Access to HIV counseling and testing among people who inject drugs in Central Asia: Strategies for improving access and linkages to treatment and care. Drug Alcohol Depend.

[10] Godinho J, Renton A, Vinogradov V, Novotny T, Rivers MJ (2005). Reversing the tide: Priorities for HIV/AIDS prevention in Central Asia.

[11] Rhodes T, Simic M (2005). Transition and the HIV risk environment. BMJ.

[12] Link BG, Phelan JC (2006). Stigma and its public health implications. Lancet.

[13] Rechel B, Sikorskaya I, McKee M (2009). Hope for health in Turkmenistan?. Lancet.

[14] Rechel B, McKee M (2007). The effects of dictatorship on health: The case of Turkmenistan. BMC Med.

[15] Boltaev AA, El-Bassel N, Deryabina AP (2013). Scaling up HIV prevention efforts targeting people who inject drugs in Central Asia: A review of key challenges and ways forward. Drug Alcohol Depend.

[16] Gupta GR, Parkhurst JO, Ogden JA, Aggleton P, Mahal A (2008). Structural approaches to HIV prevention. Lancet.

[17] Markosyan KM, Babikian T, DiClemente RJ, Hirsch JS, Grigoryan S, del Rio C (2007). Correlates of HIV risk and preventive behaviors in Armenian female sex workers. AIDS Behav.

[18] Lang DL, Salazar LF, DiClemente RJ, Markosyan K (2013). Gender based violence as a risk factor for HIV-associated risk behaviors among female sex workers in Armenia. AIDS Behav.

[19] Johnston L, Grigoryan S, Papoyan A, Grigoryan T, Balayan T, Zohrabyan L (2014). High HIV and HCV and the unmet needs of people who inject drugs in Yerevan, Armenia. Int J Drug Policy.

[20] Nassibov R, Abdukkayev A (2005). Rapid assessment on spread of injection drug use related HIV/AIDS in Azerbaijan. Int J Drug Policy.

[21] Botros BA, Aliyev QM, Saad MD (2009). HIV infection and associated risk factors among long-distance truck drivers travelling through Azerbaijan. Int J STD AIDS.

[22] Otiashvili D, Kirtadze I, O’Grady KE (2013). Access to treatment for substance-using women in the Republic of Georgia: Socio-cultural and structural barriers. Int J Drug Policy.

[23] Tsereteli N, Chikovani I, Chkhaidze N, Goguadze K, Shengelia N, Rukhadze N (2013). HIV testing uptake among female sex workers and men who have sex with men in Tbilisi, Georgia. HIV Med.

[24] Viale BN (2010). How perceived barriers to voluntary counseling & testing impact actual HIV testing among injection drug users in Kazakhstan.. http://hdl.handle.net/10211.10/624.

[25] El-Bassel N, Gilbert L, Terlikbayeva A (2014). HIV risks among injecting and non-injecting female partners of men who inject drugs in Almaty, Kazakhstan: Implications for HIV prevention, research, and policy.. Int J Drug Policy.

[26] El-Bassel N, Gilbert L, Terlikbayeva A (2011). Implications of mobility patterns and HIV risks for HIV prevention among migrant market vendors in Kazakhstan. Am J Public Health.

[27] El-Bassel N, Gilbert L, Terlikbayeva A (2013). HIV among injection drug users and their intimate partners in Almaty, Kazakhstan. AIDS Behav.

[28] Berry M, Wirtz AL, Janayeva A (2012). Risk factors for HIV and unprotected anal intercourse among men who have sex with men (MSM) in Almaty, Kazakhstan. PLoS One.

[29] Boltaev AA, Deryabina AP, Kusainov A, Howard AA (2012). Evaluation of a pilot medication-assisted therapy program in Kazakhstan: successes, challenges, and opportunities for scaleup.. Adv Prev Med.

[30] Deryabina A (2011). Mapping of key HIV services, assessment of their quality, and analysis of gaps and needs of most-at-risk populations in Chui Oblast and Bishkek City, Kyrgyzstan.. http://pdf.usaid.gov/pdf_docs/pnaea608.pdf.

[31] Messner L, Kazantseva T (2013). Gender assessment: Access to HIV services by key populationis in Kyrgyzstan.. https://www.encompassworld.com/sites/default/files/aidstarone_report_gender_assessment_web.pdf.

[32] Spicer N, Bogdan D, Brugha R, Harmer A, Murzalieva G, Semigina T (2011). ‘It’s risky to walk in the city with syringes’: understanding access to HIV/AIDS services for injecting drug users in the former Soviet Union countries of Ukraine and Kyrgyzstan. Global Health.

[33] Tkatchenko-Schmidt E, Renton A, Gevorgyan R, Davydenko L, Atun R (2008). Prevention of HIV/AIDS among injecting drug users in Russia: opportunities and barriers to scaling-up of harm reduction programmes. Health Policy.

[34] Bobrova N, Rhodes T, Power R (2006). Barriers to accessing drug treatment in Russia: A qualitative study among injecting drug users in two cities. Drug Alcohol Depend.

[35] Sarang A, Rhodes T, Platt L (2008). Access to syringes in three Russian cities: Implications for syringe distribution and coverage. Int J Drug Policy.

[36] King EJ, Maman S (2013). Structural barriers to receiving health care services for female sex workers in Russia. Qual Health Res.

[37] King EJ, Maman S, Bowling JM, Moracco KE, Dudina V (2013). The influence of stigma and discrimination on female sex workers’ access to HIV services in St. Petersburg, Russia.. AIDS Behav.

[38] Vasquez C, Lioznov D, Nikolaenko S (2013). Gender disparities in HIV risk behavior and access to health care in St. Petersburg, Russia.. AIDS Patient Care STDS.

[39] Sarang A, Rhodes T, Sheon N (2013). Systemic barriers accessing HIV treatment among people who inject drugs in Russia: A qualitative study. Health Policy Plan.

[40] Zabrocki C, Weine S, Chen S (2013). Socio-structural barriers, Protective factors, and HIV risk among Central-Asian female migrants in Moscow.. Cent Asian J Glob Health.

[41] Niccolai LM, Toussova OV, Verevochkin SV, Barbour R, Heimer R, Kozlov AP (2010). High HIV prevalence, suboptimal HIV testing, and low knowledge of HIV-positive serostatus among injection drug users in St. Petersburg, Russia.. AIDS Behav.

[42] Kruse GR, Barbour R, Heimer R (2009). Drug choice, spatial distribution, HIV risk, and HIV prevalence among injection drug users in St. Petersburg, Russia.. Harm Reduct J.

[43] Amirkhanian YA, Kelly JA, McAuliffe TL (2003). Psychosocial needs, mental health, and HIV transmission risk behavior among people living with HIV/AIDS in St Petersburg, Russia. AIDS.

[44] Amirkhanian YA, Kuznetsova AV, Kelly JA (2011). Male labor migrants in Russia: HIV risk behavior levels, contextual factors, and prevention needs. J Immigr Minor Health.

[45] Weine S, Bahromov M, Mirzoev A (2008). Unprotected Tajik male migrant workers in Moscow at risk for HIV/AIDS. J Immigr Minor Health.

[46] Stachowiak JA, Tishkova FK, Strathdee SA (2006). Marked ethnic differences in HIV prevalence and risk behaviors among injection drug users in Dushanbe, Tajikistan, 2004. Drug Alcohol Depend.

[47] Beyrer C, Patel Z, Stachowiak JA (2009). Characterization of the emerging HIV type 1 and HCV epidemics among injecting drug users in Dushanbe, Tajikistan. AIDS Res Hum Retroviruses.

[48] Weine S, Bahromov M, Loue S, Owens L (2012). Trauma exposure, PTSD, and HIV sexual risk behaviors among labor migrants from Tajikistan. AIDS Behav.

[49] Golobof A, Weine S, Bahromov M, Luo J (2011). The roles of labor migrants’ wives in HIV/AIDS risk and prevention in Tajikistan. AIDS Care.

[50] Jing L, Weine S, Bahromov M, Golobof A (2012). Does powerlessness explain elevated HIV risk amongst Tajik labor migrants? An ethnographic study. J HIV AIDS Soc Serv.

[51] Mimiaga MJ, Safren SA, Dvoryak S, Reisner SL, Needle R, Woody G (2010). “We fear the police, and the police fear us”: Structural and individual barriers and facilitators to HIV medication adherence among injection drug users in Kiev, Ukraine. AIDS Care.

[52] Booth RE, Dvoryak S, Sung-Joon M (2013). Law enforcement practices associated with HIV infection among injection drug users in Odessa, Ukraine. AIDS Behav.

[53] Todd CS, Alibayeva G, Khakimov MM, Sanchez JL, Bautista CT, Earhart KC (2007). Prevalence and correlates of condom use and HIV testing among female sex workers in Tashkent, Uzbekistan: Implications for HIV transmission. AIDS Behav.

[54] Sanchez JL, Todd CS, Bautista CT (2006). High HIV prevalence and risk factors among injection drug users in Tashkent, Uzbekistan, 2003–2004. Drug Alcohol Depend.

[55] Rechel B (2010). HIV/AIDS in the countries of the former Soviet Union: Societal and attitudinal challenges. Cent Eur J Public Health.

[56] Smolak A (2010). Contextual factors influencing HIV risk behaviour in Central Asia. Cult Health Sex.

[57] Todrys KW, Amon JJ (2009). Within but without: Human rights and access to HIV prevention and treatment for internal migrants. Global Health.

[58] Dershem L, Tabatadze M, Sirbiladze T, Tavzarashvili L, Tsagareli T, Todadze K (2007). Characteristics, high-risk behaviors and knowledge of STI/HIV/AIDS, and prevalence of HIV, syphilis and hepatitis among injecting drug users in Batumi, Georgia: 2004–2006. http://pdf.usaid.gov/pdf_docs/Pnadk406.pdf.

[59] Renton A, Gzirishvilli D, Gotsadze G, Godinho J (2006). Epidemics of HIV and sexually transmitted infections in Central Asia: Trends, drivers and priorities for control. Int J Drug Policy.

[60] Zabransky T, Mravcik V, Talu A, Jasaitis E (2014). Post-Soviet Central Asia: A summary of the drug situation. Int J Drug Policy.

[61] Bobrova N, Sarang A, Stuikyte R, Lezhentsev K (2007). Obstacles in provision of anti-retroviral treatment to drug users in Central and Eastern Europe and Central Asia: A regional overview. Int J Drug Policy.

[62] El-Bassel N, Strathdee SA, El Sadr WM (2013). HIV and people who use drugs in central Asia: Confronting the perfect storm. Drug Alcohol Depend.

[63] Estcourt CS, Marks C, Rohrsheim R, Johnson AM, Donovan B, Mindel A (2000). HIV, sexually transmitted infections, and risk behaviours in male commercial sex workers in Sydney. Sex Transm Infect.

[64] Kilmarx PH, Limpakarnjanarat K, Mastro TD (1998). HIV-1 seroconversion in a prospective study of female sex workers in northern Thailand: Continued high incidence among brothel-based women. AIDS.

[65] Hamama L, Tartakovsky E, Eroshina K (2014). Nurses’ job satisfaction and attitudes towards people living with HIV/AIDS in Russia. Int Nurs Rev.

[66] Smolak A, El-Bassel N (2013). Multilevel stigma as a barrier to HIV testing in Central Asia: A context quantified. AIDS Behav.

[67] Wolfe D, Carrieri MP, Shepard D (2010). Treatment and care for injecting drug users with HIV infection: A review of barriers and ways forward. Lancet.

[68] Wolfe D (2007). Paradoxes in antiretroviral treatment for injecting drug users: access, adherence and structural barriers in Asia and the former Soviet Union. Int J Drug Policy.

